# Prognostic value of CMR and SPECT in suspected coronary heart disease: long term follow-up of the CE-MARC study

**DOI:** 10.1186/1532-429X-18-S1-O60

**Published:** 2016-01-27

**Authors:** John P Greenwood, Bernhard A Herzog, Julia Brown, Colin C Everett, Jane Nixon, Petra Bijsterveld, Neil Maredia, Manish Motwani, Catherine Dickinson, Stephen G Ball, Sven Plein

**Affiliations:** 1 grid.418161.b0000000100972705Cardiology, Leeds General Infirmary, Leeds, UK; 2 grid.9909.90000000419368403Leeds University, Leeds, UK

## Background

CE-MARC established the comparative diagnostic performance of cardiovascular magnetic resonance (CMR) and single photon emission computed tomography (SPECT) in patients with suspected coronary heart disease. However, there are no prospective, prognostic data comparing the two modalities in the same patient population. Our objective was to establish the comparative ability of CMR and SPECT to predict major adverse cardiovascular events.

## Methods

Design: Predefined analysis of the CE-MARC trial with all patients undergoing annual follow-up for at least 5 years to assess the occurrence of major adverse cardiovascular events (cardiovascular death, acute coronary syndrome, unscheduled revascularization or hospital admission for any cardiovascular cause).

Setting: Secondary and tertiary care cardiology services.

Participants: 752 patients from CE-MARC who were under investigation for suspected coronary heart disease by a cardiologist.

Measurements: Prediction of time to major adverse cardiovascular events was assessed by univariate (log-rank test) and multivariate (Cox proportional hazards regression) analysis after adjustment for major cardiovascular risk factors. In addition net reclassification improvement (NRI) and integrated discrimination improvement (IDI) assessed whether the addition of CMR or SPECT to major cardiovascular risk factors improved the prediction of the risk of major adverse cardiovascular events.

## Results

744(99%) of 752 patients recruited had complete follow-up. Of 633 who underwent both CMR and SPECT, 105(16.6%) had at least 1 major adverse cardiovascular event. Abnormal CMR (HR 2.77; 95%CI, 1.85-4.16; p < 0.0001) and abnormal SPECT (HR 1.63; 95%CI, 1.11-2.39; p = 0.013) were both strong and independent predictors of major adverse cardiovascular events. Only CMR remained a significant predictor after adjustment for other cardiovascular risk factors and only CMR showed improved reclassification and risk stratification (NRI 0.51(95%CI 0.26-0.76); IDI 0.02(95%CI 0.005-0.05)).

## Conclusions

Five-year follow-up of CE-MARC demonstrates that only abnormal CMR added to prediction of risk of major adverse cardiovascular events beyond traditional clinical cardiovascular risk factors. This further supports the role of CMR as an alternative to SPECT for the diagnosis and management of patients with suspected coronary heart disease.

**Figure 1:**
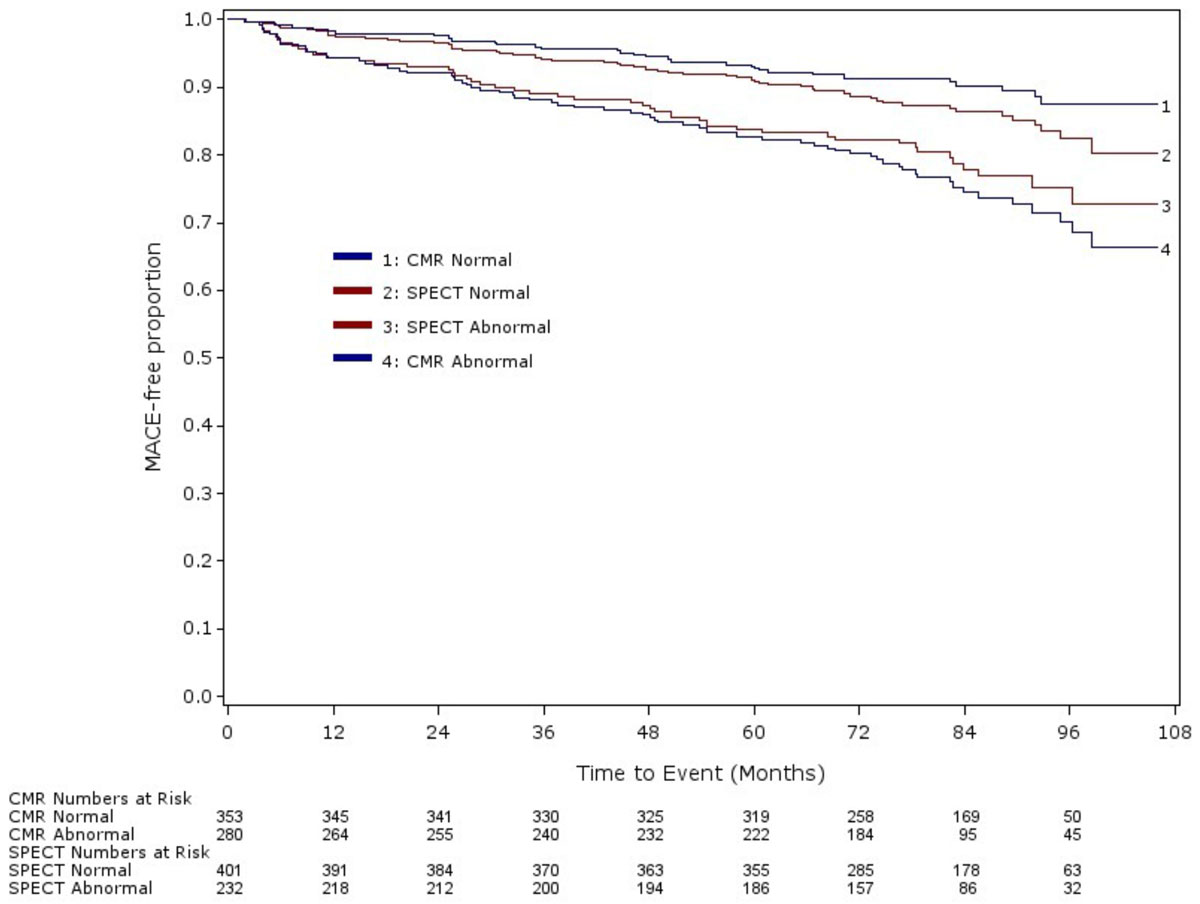
**Kaplan-Meier event curves for MACE by modality (CMR and SPECT)**.

